# Research Communication: Effects of Screening Colonoscopy on Colorectal Cancer Mortality: Lessons From Comparative Analyses of Randomised Trials

**DOI:** 10.1111/apt.70231

**Published:** 2025-06-10

**Authors:** Hermann Brenner, Dmitry Sergeev, Thomas Heisser, Michael Hoffmeister

**Affiliations:** ^1^ Division of Clinical Epidemiology and Aging Research German Cancer Research Center (DKFZ) Heidelberg Germany; ^2^ German Cancer Consortium (DKTK), German Cancer Research Center (DKFZ) Heidelberg Germany; ^3^ Heidelberg Medical Faculty Heidelberg University Heidelberg Germany

**Keywords:** colonoscopy, colorectal cancer, mortality, screening

## Abstract

NordICC, the first randomised trial on long‐term effects of screening colonoscopy, failed to demonstrate a significant reduction in colorectal cancer (CRC) mortality. We compared reported 10‐year CRC mortality results from NordICC with those of NORCCAP, a similarly designed pragmatic sigmoidoscopy trial. In NORCCAP, differences in CRC mortality only began to emerge after 9.5 years of follow‐up, rapidly increased thereafter and were approximately 4‐fold as high after 12 years than after 10 years. Other sigmoidoscopy trials yielded similar 11‐ to 12‐year results. Our analysis suggests that the apparent negative CRC mortality results of NordICC primarily reflect insufficient follow‐up time.

## Graphical abstract

The NordICC trial reported a weak non‐significant reduction of colorectal cancer mortality within 10 years from the offer of screening colonoscopy. Comparison of results with those of NORCCAP, a similarly designed randomised trial on screening sigmoidoscopy, suggests that longer follow‐up is needed for meaningful evaluation of screening colonoscopy effects.

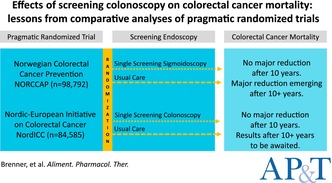

## 1 Introduction

In October 2022, results on colorectal cancer (CRC) incidence and mortality reduction by the offer of a single screening colonoscopy were reported for the first time from a randomised controlled trial (RCT), the Nordic‐European Initiative on Colorectal Cancer (NordICC) trial [1]. After a median follow‐up of 10 years, a significant reduction of CRC incidence of 18% was reported, even though the reported effect may have been underestimated for various reasons, such as delayed cancer ascertainment, as previously pointed out elsewhere [2, 3, 4]. Regarding CRC mortality, a statistically non‐significant reduction of 10% was reported. This result raised major concerns regarding the effectiveness of screening colonoscopy [5], which had previously been considered as a particularly effective preventive measure based on consistent findings from observational epidemiological studies [6]. Here, we demonstrate that the reported CRC mortality results of the NordICC trial probably underestimated the true impact of screening colonoscopy due to insufficient follow‐up time.

## 2 Methods

Our analysis is based on a comparison of the NordICC trial results on CRC mortality with those of four trials on screening sigmoidoscopy, which were reported after longer periods of follow‐up [7, 8, 9, 10]. We selected the most comparable of these, the Norwegian Colorectal Cancer Prevention (NORCCAP) trial, for a more detailed illustration of our point [10], but comparisons are also made with the results of the other sigmoidoscopy trials [7, 8, 9].

Like NordICC, NORCCAP had used a ‘pragmatic RCT design’ with a selection of study participants directly from population registries and follow‐up through death and cancer registries. It was also conducted in Norway, one of the three countries contributing to NordICC. Table 1 provides a comparison of key characteristics of NORCCAP and NordICC and their publications in 2014 and 2022. Both were designed as pragmatic RCTs with registry‐based selection and follow‐up of participants. Overall numbers (98,792 and 84,585) and age range (50–64 and 55–64) were similar. Whereas recruitment for NORCCAP was restricted to Norway, participants in NordICC were recruited from Poland (64.5%), Norway (31.2%) and Sweden (4.3%). Recruitment was performed in 1999–2001 in NORCCAP, which offered a single screening sigmoidoscopy to all invited people and an additional faecal occult blood test to 50%. NordICC recruited participants in 2009–2014 and offered a single screening colonoscopy to those randomised in the invited group. Screening adherence in the invited group was higher in NORCCAP (63%) than in NordICC (42%), but adherence in the Norwegian arm of NordICC (61%) was similar to adherence in NORCCAP. By the closing date of follow‐up for the 2014 NORCCAP report (31 December 2011), median follow‐up time for CRC mortality was 10.9 years. In the NordICC trial report, the closing date of follow‐up was not reported, but the median follow‐up was reported as 10 years.

**TABLE 1:** Summary and comparison of key characteristics and CRC mortality results obtained by intention‐to‐screen analysis of the NORCCAP trial [10] and the NordICC trial [1].

Study	NORCCAP^a^	NordICC
Design	Pragmatic RCT	Pragmatic RCT
Intervention	Single screening sigmoidoscopy, 50% + FOBT	Single screening colonoscopy
Primary end points	CRC incidence and mortality	Risk and death from CRC
Secondary endpoints	Site‐specific CRC incidence and mortality	Death from any cause
Years of recruitment	1999–2001	2009–2014
Study population		
Age range, inclusion criteria	50–64 (> 75% of CRC deaths 55–64)	55–64, no previous screening
Exclusion criteria	Death, CRC diagnosis or emigration before trial entry	Death or CRC diagnosis before trial entry
*N* invited group	20,572	28,220
*N* control group	78,220	56,365
Countries	Norway	Poland, Norway, Sweden
Screening adherence	63% (19.5% of attenders followed up by colonoscopy)	42% (Norway: 61%)
Study entry date	Proposed date of screening^b^	Date of randomization^c^
Closing date of follow‐up	31 December 2011	Not reported
Median follow‐up	10.9 years	10 years
Year of publication	2014	2022
CRC deaths (*n* [cumulative risk])		
Invited group	71 (0.34%^d^)	72 (0.28%)
Control group	330 (0.47%^d^)	157 (0.31%)
CRC mortality risk ratio (95% CI)		
Age 50–64	0.73 (0.56 to 0.94)	n.a.
Age 50–54	0.74 (0.40 to 1.35)	n.a.
Age 55–64	0.73 (0.55 to 0.97)	0.90 (0.64 to 1.16)
CRC mortality risk difference (95% CI) [percentage points]	−0.13 to (−0.22 to −0.03)^d^	−0.03 (−0.11 to 0.05)

Abbreviations: CRC, colorectal cancer; FOBT faecal occult blood test; n.a., not applicable; NORCCAP, Norwegian Colorectal Cancer Prevention Trial; NordICC, Nordic‐European Initiative on Colorectal Cancer; RCT, randomised controlled trial.

^a^ Even though further sex‐specific results were later reported from NORCCAP with extended follow‐up, we focused on the 2014 report, which, like NordICC, presented results for both sexes combined.

^b^ For participants in the control group: entry dates evenly distributed over the screening period.

^c^ Eligible population for randomization was updated every 3 to 6 months.

^d^ Not directly reported by Holme et al. [10] but approximated from reported mortality rate per 100,000 person‐years and median length of follow‐up, which was 31.4 in the invited group and 43.1 in the control group.

Based on cumulative CRC mortality curves in the invited group and the control group, which had been included in the 2014 report of the NORCCAP study [10], we assessed the effect size that would have been expected after 10 years of follow‐up compared to the effect sizes after follow‐up for up to 13 years.

## 3 Results

Reported risk ratios (95% CI) for CRC mortality from NORCCAP and NordICC are in Table 1. Similar CRC mortality risk ratios as for NORCCAP have been reported after 11 to 12 years of follow‐up in all four screening sigmoidoscopy RCTs, with relative risk (95% CI) estimates ranging from 0.69 (0.59–0.82) to 0.78 (0.56–1.08) (Table S1).

**FIGURE 1:**
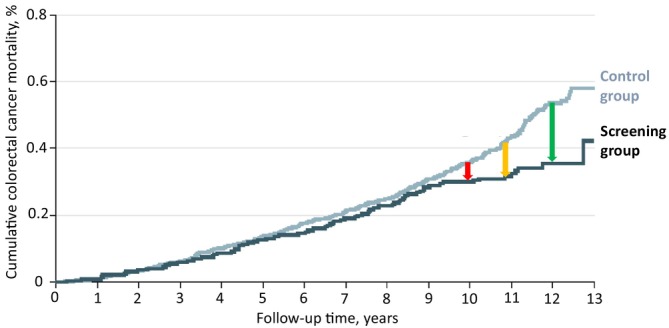
Cumulative colorectal cancer mortality risk within 13 years of follow‐up in the NORCCAP trial (extracted from Holme et al. [10]), and absolute risk reductions in the screening group seen after 10 years (red arrow), 10.9 years (yellow arrow) and 12 years (green arrow) of follow‐up.

Figure 1 shows the time course of cumulative CRC mortality in NORCCAP during up to 13 years of follow‐up [10]. After 10 years of follow‐up (the median in NordICC), a modest reduction in cumulative CRC mortality by roughly 0.0005 (0.05%) would have been observed (red arrow). By contrast, after 10.9 and 12 years of follow‐up, an almost 2‐fold and 4‐fold higher mortality reduction was seen in NORCCAP (yellow and green arrow, respectively).

## 4 Discussion

Publication of preliminary results after 10 years of follow‐up of NordICC has prompted major uncertainty and doubts regarding screening colonoscopy for reducing CRC incidence and mortality. In particular, the statistically non‐significant reduction of CRC mortality has been perceived as disappointing, given that observational epidemiological studies had consistently found much greater reductions [5]. RCTs are generally considered less prone to bias than observational epidemiological studies. Therefore, this apparent discrepancy has been widely interpreted as rectification of bias by the observational studies through the first RCT. However, our analysis suggests that, besides lower‐than‐expected adherence, insufficient follow‐up time may be a major reason for the failure of NordICC to confirm the expected effects of screening colonoscopy on CRC mortality.

In NORCCAP, major effects of screening by a single sigmoidoscopy (partly combined with a faecal occult blood test) started to emerge only after 10 years of follow‐up, even though the entry date of follow‐up started with the proposed screening date, later than in NordICC (where it started with the date of randomization). In combination with lower screening adherence in NordICC than in NORCCAP (42% versus 63%), the apparent null results of NordICC regarding CRC mortality are therefore not surprising.

Effective follow‐up time in NordICC might have been even shorter than reported. In NORCCAP, results after a median of 10.9 years' follow‐up were reported in 2014 (13–15 years after recruitment). In NordICC, results after a median 10 years' follow‐up were reported in 2022 (8–13 years after recruitment). Taking into account delays in completeness of cause‐specific mortality registration and the time needed for data work‐up and analysis and for manuscript preparation and review, 10‐year follow‐up may have been incomplete for a proportion of the NordICC cohort by the time the data were analysed, raising concerns about the completeness of 10‐year mortality follow‐up.

We focused our comparative analysis on NORCCAP as the most comparable sigmoidoscopy trial, and due to inclusion of participants from Norway. Both trials recruited participants directly identified from population registries and followed them by record linkage with cancer and death registries. By contrast, participants more likely to adhere had been pre‐selected in the sigmoidoscopy trials in the United Kingdom and Italy [7, 8]. The PLCO trial from the United States additionally offered a second screening sigmoidoscopy after 5 years and included participants within a broader age range [9]. Notwithstanding these differences, effect estimates on CRC mortality after 11–12 years of follow‐up were overall very similar and consistently stronger in all sigmoidoscopy trials than those reported from NordICC, with most mortality reduction emerging in later years of follow‐up.

Even though adherence to screening was lower in NordICC (42%) than in the sigmoidoscopy trials (≥ 58%), this difference is insufficient to explain the apparent much lower reduction of CRC mortality in the intention‐to‐treat analyses in NordICC. Furthermore, by examination of the entire colon and rectum, even stronger effects of screening colonoscopy would have been expected.

Differences between NordICC and NORCCAP require careful discussion. First, NORCCAP also included participants aged 50–54 years. However, reported hazard ratios were almost identical in the 50–54 and 55–64 year age groups (0.73 and 0.74, respectively). Second, only approximately one third of NordICC participants came from Norway, and screening effects were substantially weaker in the majority of participants recruited from Poland [1]. Along with the lower screening adherence rate in Poland, this may be another reason for the lower effect estimate for screening colonoscopy in NordICC compared to screening sigmoidoscopy in NORCCAP, despite the expected stronger effects of screening colonoscopy. Third, NordICC participants were recruited approximately 10 years later than NORCCAP participants. Progress in CRC therapy and prognosis or changes in risk factor profiles might have led to reductions in CRC mortality even in the absence of screening in more recent years.

Notwithstanding the differences in endoscopy, the temporal patterns of manifestation of screening effects observed for screening sigmoidoscopy would not be expected to be fundamentally different for screening colonoscopy. Despite the limitations in trial comparability, our analyses therefore support concerns that the reported results from the NordICC trial on CRC mortality need to be interpreted with caution, as they may have underestimated the true impact of screening colonoscopy due to insufficient follow‐up time. Results published to date should not discourage widespread use of CRC screening, which went along with tremendous reductions of CRC incidence and mortality in the screening age groups (in contrast to rising incidence rates at younger ages) in several countries including the US in the past decades.

## Author Contributions

**Hermann Brenner:** conceptualization, writing – original draft, writing – review and editing. **Dmitry Sergeev:** writing – review and editing. **Thomas Heisser:** writing – review and editing. **Michael Hoffmeister:** writing – review and editing.

## Conflicts of Interest

The authors declare no conflicts of interest.

## Supporting information


**Table S1.** Effect estimates of the offer of screening sigmoidoscopy on colorectal cancer mortality reported after 11 to 12 years of follow‐up from the four sigmoidoscopy‐based randomised trials.

## Data Availability

The authors have nothing to report.
