# Supplementary material for: Research Communication: Effects of Screening Colonoscopy on Colorectal Cancer Mortality: Lessons From Comparative Analyses of Randomised Trials
Source: Aliment Pharmacol Ther. 2025 Jun 10;62(6):656–9. doi: 10.1111/apt.70231 (PMC12395887; doi:10.1111/apt.70231)
Supplement: Supplementary file 1 — Table S1. Effect estimates of the offer of screening sigmoidoscopy on colorectal cancer mortality reported after 11 to 12 years of follow‐up from the four sigmoidoscopy‐based randomised trials. [file APT-62-656-s001.docx]

**Supplementary Table 1.** Effect estimates of the offer of screening sigmoidoscopy on colorectal cancer mortality reported after 11 to 12 years of follow-up from the four sigmoidoscopy-based randomized trials

| First author, year | Trial | Country | Age range | Median follow-up time | Relative risk (95% CI) |
| --- | --- | --- | --- | --- | --- |
|  |  |  |  |  |  |
| Atkin, 2010 (7) | UKFSST | UK | 55-64 | 11.2 years | 0.69 (0.59-0.82) |
| Segnan, 2011 (8) | SCORE | Italy | 55-64 | 11.4 years | 0.78 (0.56-1.08) |
| Schoen, 2012 (9) | PLCO | US | 50-64 | 11.9 years | 0.74 (0.63-0.87) |
| Holme, 2014 (10) | NORCCAP | Norway | 55-74 | 10.9 years | 0.73 (0.56-0.94) |

NORCCAP, Norwegian Colorectal Cancer Prevention Trial; PLCO, Prostate, Lung, Colorectal, and Ovaria Cancer Screening Trial; SCORE, Screening for COlon REctum; UKFSST, United Kingdom Flexible Sigmoidoscopy Screening Trial
